# A Deoxyuridine-Based Far-Red Emitting Viscosity Sensor

**DOI:** 10.3390/molecules21060709

**Published:** 2016-05-30

**Authors:** Mengyuan Wang, Yuanwei Zhang, Xiling Yue, Sheng Yao, Mykhailo V. Bondar, Kevin D. Belfield

**Affiliations:** 1 Department of Chemistry, University of Central Florida, P.O. Box 162366, Orlando, FL 32816, USA; wmyround@knights.ucf.edu (M.W.); zhangyuanwei2008@hotmail.com (Y.Z.); xyue@sbpdiscovery.org (X.Y.); sheng.yao@ucf.edu (S.Y.); 2 Institute of Physics, National Academy of Sciences of Ukraine, Prospect Nauki, 46, Kiev-28 03028, Ukraine; mike_bondar@hotmail.com; 3 School of Chemistry and Chemical Engineering, Shaanxi Normal University, Xi’an 710062, China; 4 College of Science and Liberal Arts, New Jersey Institute of Technology, University Heights, Newark, NJ 07102, USA

**Keywords:** microviscosity sensor, far-red fluorescent probe, bioimaging, nucleosides, Sonogashira coupling, molecular rotor

## Abstract

A novel deoxyuridine (dU) benzothiazolium (BZ) derivative, referred to as dU-BZ, is reported that was synthesized via Sonogashira coupling reaction methodology. The deoxyuridine building block was introduced to enhance hydrophilicity, while an alkynylated benzothiazolium dye was incorporated for long wavelength absorption to reduce potential phototoxicity that is characteristic of using UV light to excite common fluorphores, better discriminate from native autofluorescence, and potentially facilitate deep tissue imaging. An impressive 30-fold enhancement of fluorescence intensity of dU-BZ was achieved upon increasing viscosity. Fluorescence quantum yields in 99% glycerol/1% methanol (*v*/*v*) solution as a function of temperature (293–343 K), together with viscosity-dependent fluorescence lifetimes and radiative and non-radiative rate constants in glycerol/methanol solutions (ranging from 4.8 to 950 cP) were determined. Both fluorescence quantum yields and lifetimes increased with increased viscosity, consistent with results predicted by theory. This suggests that the newly-designed compound, dU-BZ, is capable of functioning as a probe of local microviscosity, an aspect examined by *in vitro* bioimaging experiments.

## 1. Introduction

Information, such as physiological composition, can be reflected by microenvironments in cellular compartments [1]. For example, an increase in mitochondrial membrane viscosity was discovered after exposure to β-amyloid, which is essentially involved in Alzheimer′s disease [2]. Mechanical methods [3,4] have been universally applied to measure the viscosity of bulk liquids. However, viscosity on the microscopic scale may differ largely. It is a significant challenge to use techniques to measure microviscosity on the order of micrometers so that intracellular viscosity can be probed.

One method to monitor viscosity changes at the single cell level is the use of fluorescence imaging with molecular rotors [5,6]. Molecular rotors are fluorophores whose fluorescence intensity is affected by intramolecular rotation that can be greatly affected by the viscosity of its surrounding environment. This can be accomplished via an intramolecular charge transfer (ICT) mechanism by molecular twisting in the excited state. Two excited states, a local excited (LE) state and twisted intramolecular charge transfer (TICT) state, are involved [7], and de-excitation to the ground state can occur from both states. With intramolecular twisting at in the excited state, different molecular conformations lead to an energy gap between the LE state and TICT state, then non-radiative deactivation can occur from the LE state to a dark, non-emissive TICT state. This energy gap leads to different intensities of radiative decay from two excited states to the ground state. In viscous media, the rate constant of non-radiative relaxation is reduced, the radiative decay of the LE state occurs, and the quenched emission by de-excitation from TICT state is recovered, resulting in a higher fluorescence quantum yield and longer fluorescence lifetime [8].

Molecular rotors have been proposed over the decades for measurement of local viscosity by tracking the changes in fluorescence quantum yields [6,9]. A challenge in this approach is separating influences on fluorescence intensity caused by viscosity from other factors, such as local concentration of fluorophores and specific solvent effects. A ratiometric approach was applied to address this problem [5,10,11,12]. By conjugating the molecular rotor to a fluorescent label whose quantum yield is not affected by viscosity, the concentration can be determined in different viscous environments [13,14]. An alternative method to determine viscosity by molecular rotors is the application of fluorescence lifetime imaging microscopy, since the fluorescence lifetime of a molecular rotor does not change with the concentration of the fluorophore, but changes with fluorescence quantum yield as a function of viscosity [15,16]. In addition, conjugation of molecular rotors to another fluorophore can be omitted, which, in general, simplifies the synthesis and leaves the possibility for further functionalization of the probe’s structure.

Our interest is the design and synthesis of biocompatible probes emitting in the far-red region for bioimaging [17,18,19], including cyanine dyes. Augmenting the properties of organic fluorophores with biomolecules, especially nucleosides, are quite interesting [20,21,22,23]. Conjugation of fluorophores as side chains to DNA nucleosides is favorable because modified nucleosides can be paired with the complementary strand without radically altering the structure [24]. Additionally, requirements such as reduced toxicity and enhanced hydrophilicity can be fulfilled by modifying the compound with biocompatible nucleoside building blocks. Live cell uptake studies suggest that nucleoside-modified fluorophores can function as biological probes [25]. Usually carried out by Sonogashira coupling to conjugate nucleosides and fluorophores, our aim was to introduce an acetylene linker to join them, and this linker can avoid steric hindrance that often accompanies direct coupling [26]. A squaraine and deoxyuridine-based viscosity sensor, dU-SQ, was reported by our group, exhibiting a 300-fold fluorescence increase [25]. Optimizing reaction conditions for Sonagashira coupling has been carefully studied, and by utilizing amberlite IRA-67, a milder base when compared to some conventional bases such as DIPEA and TEA, much cleaner reactions were realized. Although this great increase in fluorescence intensity was due to not only TICT but also affected by aggregation of squaraine dyes, this new compound supported that intercellular viscosity is dependent on microtubules (MTs) crosslinking and density, and cell images were captured during different stages of mitosis.

Herein, to circumvent complications related to aggregation, we report a newly designed molecular rotor dU-BZ, formed via Sonogashira coupling to covalently link a cyanine chromophore to deoxyuridine through an acetylene moiety. Linear absorption, emission spectra, and fluorescence quantum yields of dU-BZ in glycerol/methanol solutions were obtained, and a 30-fold fluorescence enhancement was realized in a purely viscosity-dependent manner (no aggregation effects were observed such as those reported previously). Far-red excitation and emission ensures lower risks of photodamage and phototoxicity, should help generate signals less convoluted with autofluorescence, and may facilitate deep tissue imaging. *In vitro* fluorescence microscopy was conducted to demonstrate that the potential of this new compound as a microviscosity probe at the cellular level.

## 2. Results

### 2.1. Synthesis of dU-BZ

**Scheme 1:**
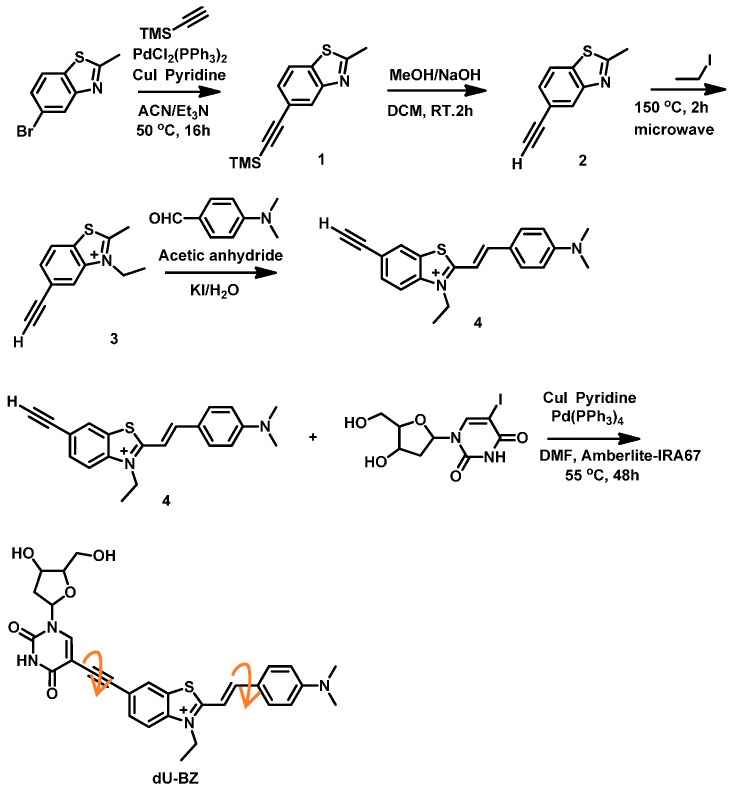
Synthetic route of molecular rotor dU-BZ. The arrow on the dU-BZ structure indicates the major molecular rotation that contributes to the fluorescence on-off feature.

The synthesis of molecular rotor dU-BZ is illustrated in Scheme 1. Intermediate compound **3** was synthesized according to the literature [25], and the resulting NMR matched the reported data. Next, compound **4** was synthesized by condensation of intermediate **3** with dimethylaminobenzaldehyde via a Knoevenagel reaction; acetic anhydride was employed as both base and solvent.

The following procedure was used for the conjugation of a deoxyuridine analog and alkynylbenzothiazolium **4** through a triple bond. Rather than directly using deoxyuridine, a modified form, idoxuridine, was exploited not only due to the iodo group provided for conjugation, but also its structure may be incorporated into DNA/RNA strands for future study. Hydroxyl groups of idoxuridine are all unprotected in order to avoid possible low overall yield. Although protected hydroxyl groups possess enhanced water solubility when compared to unprotected ones, in consideration of the overall yield of the reaction, protected nucleosides were not pursued.

dU-BZ was obtained via Sonogashira coupling between (+)-5-iodo-2′-deoxyuridine and alkyne **4** in 21% yield after purification by column chromatography. After conjugation with the deoxyuridine analog, dU-BZ exhibited enhanced water solubility when compared to **4**. The ^1^H-NMR, ^13^C-NMR, and HR-MS spectra were in good accordance with the chemical structure as expected.

### 2.2. Linear Photophsical Characterization of dU-BZ by Varying Temperature

**Figure 1:**
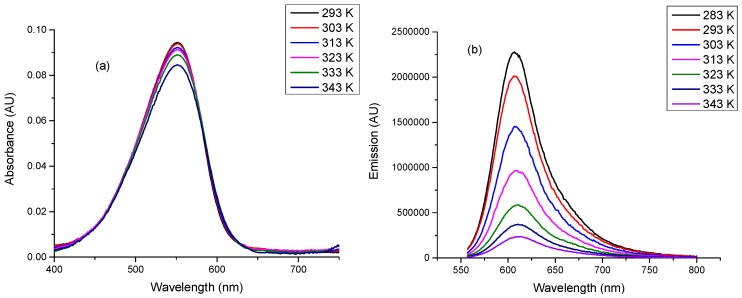
Absorption (**a**) and emission (**b**) spectra of dU-BZ recorded as a function of temperature in 99% glycerol/1% methanol (*v*/*v*) solution. Excitation wavelength = 544 nm.

The fluorescence quantum yields (Φ_f_) of dU-BZ were measured in 99% glycerol/1% methanol (*v*/*v*) solution at various temperatures, ranging from 343 to 293 K, with viscosity ranging from 50.6 to 1412 cP (Table 1). According to Figure 1a,b, no significant shifts were observed in absorption and emission spectra, but an increase in the fluorescence intensity was obtained with decreasing temperature when excited at 551 nm, and Φ_f_ increased from 0.04 to 0.34.

### 2.3. Linear Photophysical Characterization of dU-BZ as A Function of Viscosity

**Figure 2:**
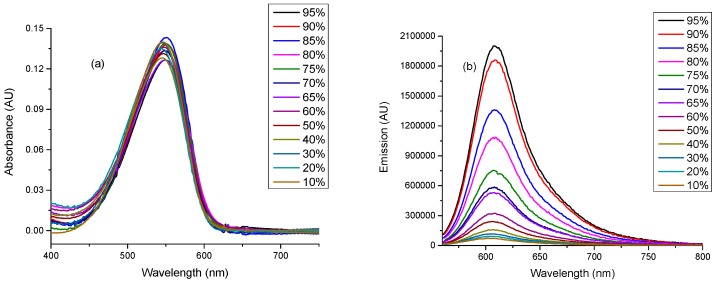
Absorption (**a**) and emission (**b**) spectra of dU-BZ obtained as a function of viscosity in glycerol/methanol (*v*/*v*) solutions; percentage indicated is the glycerol content of the solution.

At the same concentration, there was no obvious change observed in the absorption spectra for dU-BZ by varying the ratio of glycerol and methanol in solution. However, without any changes in shape of the emission spectrum or the peak emission wavelength, a 30-fold increase in fluorescence intensity appeared at 608 nm by increasing the viscosity from 1.8 cP to 950 cP (Figure 2).

### 2.4. Fluoresence Lifetime of dU-BZ in Glycerol/Methanol Solutions, Radiative, and Non-Radiative Rate Constants

**Figure 3:**
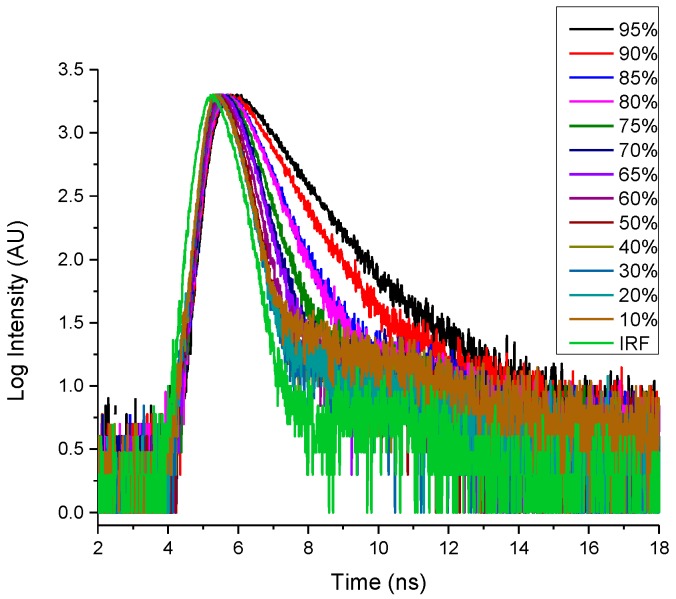
Fluorescence decay of dU-BZ recorded as a function of viscosity in glycerol/methanol solution; percentage indicated is the content of glycerol in solution.

Figure 3 shows the fluorescence lifetime decay of dU-BZ with decreasing viscosity in glycerol/methanol solutions. As a function of viscosity, the fluorescence lifetime varied markedly from 0.19 ns at 58 cP to 1.07 ns at 950 cP.

### 2.5. In Vitro Bioimaging of dU-BZ

**Figure 4:**
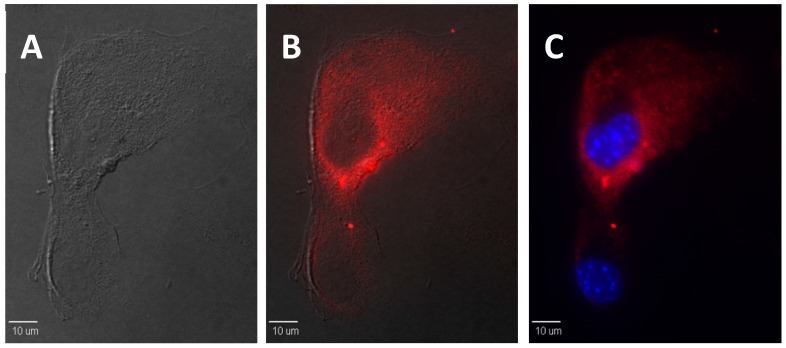
3T3 cells were incubated with dU-BZ (15 μM, 30 min). DIC image (**A**) indicates healthy morphology of 3T3 cells. Overlay image of dU-BZ fluorescence and DIC (**B**) indicates effective uptake of dU-BZ (Texas Red filter cube (Ex 562/40 nm, DM 593 nm, Em 624/40 nm). (**C**) shows overlay image of Hoechst (blue filter cube, Ex 377/50 nm, DM 409 nm, Em 460/50 nm), and dU-BZ fluorescence. Scale bar is 10 μm.

Highly viscous, up to 400 cP, intra- and intercellular environments [15] have been reported. The *in vitro* fluorescence enhancement using dU-BZ was explored, and, indeed, after incubation with 3T3 cells (mouse embryonic fibroblast cells) for 30 min, dU-BZ appeared to readily enter the cells, and remarkably clear fluorescence images were obtained (Figure 4, Hoechst stained cell nuclei as reference). These results suggest that dU-BZ can, potentially, be utilized to visualize viscous regions at the cellular level, providing motivation for further studies with this promising new probe.

## 3. Discussion

Free-volume concepts [27] can be described by the fluorescence quantum yield, Φ_f_, viscosity, η, and temperature, T [9]:

Φ_f_ = B (η/T)^x^, (1)

where B = (k_r_/k_nr0_)·(T/A)^x^, k_nr0_ is the free-rotor reorientation rate, A is a constant, and x is a medium-dependent constant ranging between 0 and 1. When Φ_f_ is linearly related to η/T (x = 1), the bulk viscosity of solvent can accurately indicate the friction experienced by the molecular rotor. Normally, faster rotational diffusion is expected because the fluorophore can occupy a certain free volume within the solvent, in which case x < 1. A plot of log Φ_f_ verses log (η/T) yields a straight line with the exponent x as its slope via Equation (2):

log Φ_f_ = x log (η/T) + x log B, (2)

**Figure 5:**
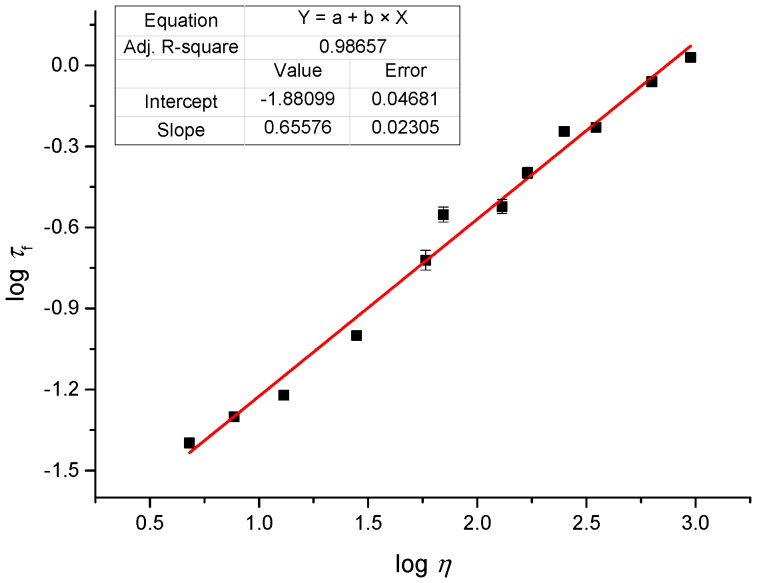
Plot of log Φ_f_ *vs*. log (η/T) for dU-BZ in 99% glycerol/1% methanol (*v*/*v*) solution.

As shown in Figure 5, linear behavior was observed when plotting log Φ_f_ *vs.* log (η/T). The slope of this plot provided the exponent x, 0.57 ± 0.04, in the range from 0 to 1, with a R^2^ value of 0.98. Due to increased viscosity and decreased free volume, a decreased non-radiative rate constant is expected, and this prediction will be described in the following experiments.

The Förster-Hoffmann equation [28] can be used to describe Φ_f_, and fluorescent lifetime, τ_f_, of molecular rotors as a function of η:

Φ_f_ = zη^α^, (3)

where:

Φ_f_ = k_r_/(k_r_ + k_nr_), (4)

τ_f_ =1/(k_r_ + k_nr_), (5)

then:

τ_f_ = zk_r_^−1^η^α^, (6)

where z and α are constants, the value of 2/3 for α is predicted by Förster and Hoffmann, and k_r_ and knr are radiative and non-radiative rate constants. [29] According to Equation (6), a straight line with a slope of α will be yielded after plotting log τ_f_ verses log η, since:

log τ_f_ = α log η + log (z/k_r_), (7)

One should note that Equation (3) can only be applied over a limited range of viscosities. According to the Förster-Hoffmann theory, Φ_f_ is solvent-independent at low viscosities, whereas at relatively high viscosities, a strong dependence on viscosity of Φ_f_ is expected, since radiative processes predominate over non-radiative relaxation. This very range of viscosities is determined by the properties of the particular molecular rotor and the mechanism of viscosity-dependent photophysical behavior.

**Figure 6:**
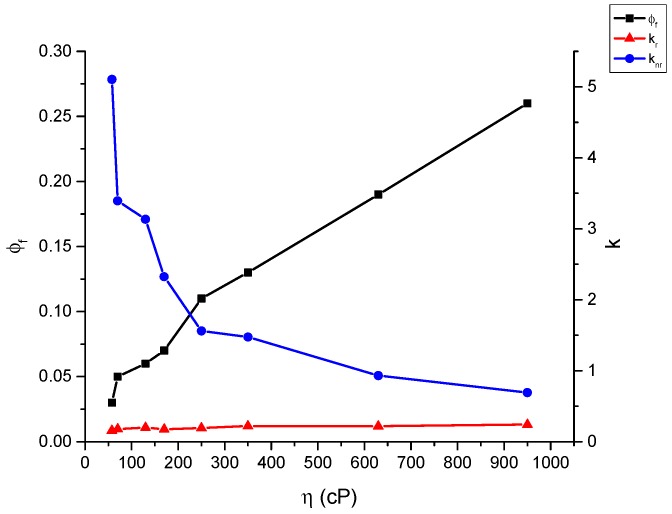
Fluorescence quantum yield (*Φ*_f_), radiative (*k*_r_), and non-radiative (*k*_nr_) rate constants of dU-BZ obtained as a function of viscosity (*η*) in glycerol/methanol solutions.

Measured values, Φ_f_ and τ_f_, were used to calculate the rate constants via Equations (2) and (3). Plotted in Figure 6, with viscosity increasing from 58 cP to 950 cP (Table 2), Φ_f_ shows significantly increased values as expected. It is worth noting that k_r_ remained constant but k_nr_ decreased largely as a function of viscosity. These data suggest that the main contribution to the increase of Φ_f_ is via suppression of the non-radiative process. In a highly viscous environment, because of the intramolecular rotation hindrance, the torsion angle between the benzothiazole and aminobenzene rings is close to zero, which yields the most stable conformation of the molecule in the LE state. At the same time, non-radiative relaxation to the TICT state, which has a conformation angle value close to 90°, is deactivated, and radiative decay from LE state to ground state starts to take place instead of de-excitation from the TICT state.

**Figure 7:**
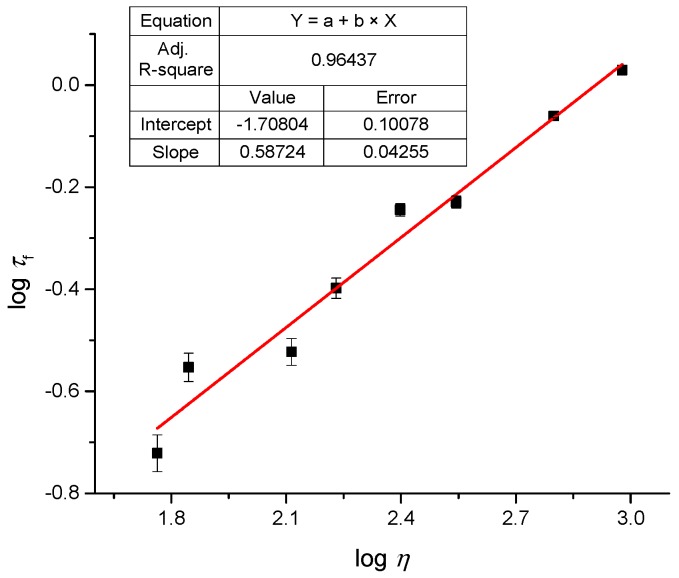
Plot of log τ_f_ *vs*. log *η* for dU-BZ.

From Equation (7), a straight line was obtained in the plot of log τ_f_ *vs*. log *η* and, as expected, linear behavior was obtained (Figure 7) with a slope, α, of 0.59 ± 0.04, consistent with the value predicted by the Förster-Hoffmann equation, and a R^2^ value of 0.96 for dU-BZ. It was also found that plots below 58 cP fit in the same straight line, but lifetime values lower than 0.2 ns were not reliable due to the resolution of the experimental system (Table 2). Only plots from 58 cP to 950 cP are shown in the figure.

## 4. Materials and Methods

### 4.1. Synthesis

Synthetic reagents and solvents were used as received from commercial suppliers. 5-Bromo-2-methylbenzothiazole was purchased from TCI America (Portland, OR, USA). Iodoethane and (+)-5-iodo-2′-deoxyuridine were purchased from Alfa Aesar (Ward Hill, MA, USA). ^1^H- and ^13^C-NMR spectra were recorded on a Bruker Avance III 400 NMR spectrometer at 400 and 101 MHz, respectively (Billerica, MA, USA). High-resolution mass spectrometry analysis was performed in the Department of Chemistry, University of Florida. Uncorrected melting points were determined using a Laboratory Devices mel-temp.

*2-Methyl-5-((trimethylsilyl)ethynyl)benzothiazole* (**1**). Under an argon atmosphere 5-bromo-2-methylbenzothiazole (1.5 g, 6.30 mmol), bis(triphenylphosphine)palladium(II) dichloride (442 mg, 0.63 mmol), copper iodide (144 mg, 0.75 mmol) were mixed in 30 mL of degassed acetonitrile and triethyl amine solution (1:1, *v*/*v*). trimethylsilylacetylene (4.50 mL) was added before stirring at room temperature for 10 min. Pyridine (3 mL) was added, and the resulting mixture was first stirred at room temperature for 30 min, then at 50 °C for 18 h. After being cooled to room temperature, solvent was removed under reduced pressure and the solid residue was purified by column chromatography (silica gel, degrade elution hexanes/ethyl acetate from 10:1 to 7:1), resulting in 1.50 g of white solid (93% yield), m.p.: 126–127.5 °C. ^1^H-NMR (400 MHz, CDCl_3_) δ: 8.02 (d, *J* = 1 Hz, 1H), 7.72 (d, *J* = 8.3 Hz, 1H), 7.43 (dd, *J* = 8.3, 1.5 Hz, 1H), 2.82 (s, 3H), 0.28 (s, 9H). ^13^C-NMR (101 Hz, CDCl_3_) δ: 167.91, 153.25, 138.06, 128.31, 125.83, 121.04, 104.77, 94.38, 20.21 ppm. HR-MS (ESI) theoretical [M + H]^+^ = 246.0767, found [M + H]^+^ = 246.0777.

*5-Ethynyl-2-methylbenzothiazole* (**2**). 2-Methyl-6-((trimethylsilyl)ethynyl)benzothiazole (1 g, 4.07 mmol) was dissolved in 15 mL of dichloromethane, and 15 mL of methanol/NaOH solution (3%, *w*/*w*) was added dropwise. The mixture was allowed to stir at room temperature for 2 h, followed by the removing the organic solvent *in vacuo*. The solid residue was further purified by column chromatography (silica gel, hexanes/ethyl acetate 10:1), affording 0.54 g of pale yellow crystal (77% yield), m.p.: 66–67 °C. ^1^H-NMR (400 MHz, CDCl_3_) δ: 8.07 (d, *J* = 1.5 Hz, 1H), 7.75 (d, *J* = 8.3 Hz, 1H), 7.45 (dd, *J* = 8.1, 1.7 Hz, 1H), 3.12 (s, 1H), 2.82 (s, 3H). ^13^C-NMR (101 Hz, CDCl_3_) δ: 168.12, 153.21, 136.39, 128.26, 126.09, 126.08, 121.33, 119.79, 83.38, 21.08 ppm. HR-MS (ESI) theoretical [M + H]^+^ = 174.0372, found [M + H]^+^ = 174.0378.

*5-Ethynyl-3-ethyl-2-methylbenzothiazolium iodide* (**3**). 5-Ethynyl-2-methylbenzothiazole (1 g, 5.78 mmol) was mixed with 2 mL of iodoethane in 1.5 mL of degassed acetonitrile. The mixture was heated in a microwave reactor (CEM, discover) at 150 °C for 20 min. Precipitate was collected by filtration and washed with diethyl ether to afford 1.06 g of grey powder, (91% yield), m.p.: 267 °C (dec.). ^1^H-NMR (400 MHz, DMSO-*d*_6_) δ: 8.54 (d, *J* = 1.1 Hz, 1H), 8.43 (dd, *J* = 8.5, 1.5 Hz, 1H), 7.86 (dd, *J* = 8.4, 1.3 Hz, 1H), 4.78 (q, *J* = 7.2 Hz, 2H), 4.60 (s, 1H), 3.21 (s, 3H), 1.44 (m, 3H). ^13^C-NMR (101 Hz, DMSO-*d*_6_) δ: 178.89, 141.14, 131.39, 130.18, 125.65, 123.20, 120.19, 84.29, 82.36, 45.36, 17.47, 13.74 ppm. HR-MS (ESI) theoretical [M]^+^ = 202.0685, found [M]^+^ = 202.0692.

*2-(4-(Dimethylamino)styryl)-3-ethyl-5-ethynylbenzothiazolium iodide* (**4**). 5-eEhynyl-3-ethyl-2-methylbenzothiazolium iodide (1.5 g, 7.42 mmol) and 2-methyl-*N*-benzaldehyde (1.33 g, 8.90 mmol) were mixed with 126 mL of acetic anhydride. The mixture was refluxed at 150 °C for 20 min and then the hot solution was poured into 200 mL of warm KI solution. After cooling to room temperature, precipitate was filtered and washed with water and a large amount of diethyl ether, yielding 1.81 g of purple solid (73% yield), m.p.: 256 °C (dec.). ^1^H-NMR (400 MHz, DMSO-*d*_6_) δ: ppm 1.37–1.44 (m, 3H) 3.04 (s, 1H) 3.13 (s, 6H) 4.54 (s, 1H) 4.78–4.85 (m, 2H) 6.86 (d, *J* = 9.05 Hz, 2H) 7.59 (d, *J* = 15.16 Hz, 1H) 7.73 (d, *J* = 8.31 Hz, 1H) 7.94 (d, *J* = 9.05 Hz, 2H) 8.11 (d, *J* = 14.92 Hz, 1H) 8.27–8.31 (m, 2H). ^13^C-NMR (101 MHz, DMSO-*d*6) δ 14.62, 40.54, 44.29, 83.01, 84.10, 100.30, 106.04, 112.74, 119.21, 122.23, 123.03, 125.12, 128.64, 134.07, 141.85, 152.08, 154.50, 172.35. HR-MS (ESI) theoretical [M]^+^ = 333.1420, found [M]^+^ = 333.1416.

*Synthesis of dU-BZ* (**5**). Under an argon atmosphere a mixture of 5-iodo-2′-deoxyuridine (425 mg, 1.2 mmol), 4 (1.2 g, 3.60 mmol), Pd(PPh_3_)_4_ (139 mg, 0.12 mmol), CuI (47 mg, 0.24 mmol), and 550 mg of Amberlite IRA-67 in 11.3 mL of degassed DMF was stirred at 55 °C for 48 h. The Amberlite IRA-67 beads were excluded by filtration first, and to the DMF solution diethyl ether was added, the resulting precipitate was collected by filtration. Further purification was carried out by column chromatography (silica gel, dichloromethane/methanol 10:1), resulting in 141 mg of purple solid (21% yield), m.p.: 249 °C (dec.). ^1^H-NMR (400 MHz, DMSO-*d*_6_) δ: ppm 1.38–1.45 (m, 3H) 2.20 (d, *J* = 6.11 Hz, 2H) 3.13 (s, 6H) 3.84 (q, *J* = 3.18 Hz, 1H) 4.25–4.31 (m, 1H) 4.80–4.90 (m, 2H) 5.23–5.37 (m, 2H) 6.14 (t, *J* = 6.36 Hz, 1H) 6.86 (d, *J* = 9.05 Hz, 2H) 7.62 (d, *J* = 15.16 Hz, 1H) 7.73 (dd, *J* = 8.44, 1.34 Hz, 1H) 7.95 (d, *J* = 9.05 Hz, 2H) 8.12 (d, *J* = 15.16 Hz, 1H) 8.23 (s, 1H) 8.32 (d, *J* = 8.31 Hz, 1H) 8.50 (s, 1H) 11.78 (br. s., 1H). ^13^C-NMR (101 MHz, DMSO-*d*_6_) δ 14.62, 31.15, 51.96, 61.57, 70.65, 85.15, 85.83, 88.37, 88.43, 98.29, 106.15, 112.82, 118.24, 122.31, 123.83, 125.13, 128.22, 128.95, 129.31, 130.83, 134.07,150.18, 152.04, 154.53, 158.08, 172.37. HR-MS (ESI) theoretical [M]^+^ = 559.2010, found [M]^+^ = 559.2006.

### 4.2. Viscosity Values

Viscosities of pure glycerol at different temperature were employed to approximate the viscosity of 99% glycerol/1% methanol solution. Reported values [30] were directly used as viscosities of solutions with glycerol percentages ranging from 10% to 95%.

### 4.3. Linear Photophysical Characterization

The linear absorption spectra were obtained using an Agilent 8453 UV−VIS spectrophotometer (Agilent, Santa Clara, CA, USA) in 10 mm path length quartz cuvettes in solvents with different glycerol/methanol ratios, with molar concentration C = 1 × 10^−5^ M. The steady-state fluorescence was measured with a PTI QuantaMaster spectrofluorimeter using 10 mm spectrofluorometric quartz cuvettes with C = 1 × 10^−6^ M. The correction for the spectral response of the PTI detection system was performed for all fluorescence spectra. The fluorescence quantum yields, Φ_f_, were obtained by a standard method [31] relative to cresyl violet in methanol. Fluorescence lifetimes, τ_f_, were measured using a PicoQuant PicoHarp 300 time-correlated single photon-counting system with time resolution ≈ 80 ps, a Coherent Mira 900 fs laser system was used for excitation, linearly polarized at the magic angle.

### 4.4. In Vitro Bioimaging

3T3 cells (ATCC^®^, Manassas, VA, USA) were seeded on poly-d-lysine coated coverslips at a concentration of 5 × 10^4^ cells/mL and incubated for 48 h. A dU-BZ stock solution in DMSO (dimethyl sulfoxide) was then diluted to 15 μM with DMEM medium (Cellgro^®^, Mediatech, Menassas, VA, USA) and added to the cells. Cells were co-incubated with diluted dU-BZ for 30 min and then fixed with 4% formaldehyde. NaBH4 was added twice at 1 mg/mL for 5 min to reduce auto-fluorescence. Cells were then permeabilized with 0.1% Triton-X. 1% BSA was applied to block non-specific binding. Hoechst 33258 (Invitrogen™, Carlsbad, CA, USA) was added in to cell for 5 min to visualize cell nuclei. Coverslips were then washed with PBS (phosphate-buffered saline, Cellgro^®^) and mounted on slides with ProLong Gold^®^ (Invitrogen™) antifade reagent (Invitrogen™).

Cells were imaged with an IX70 DSU microscope (Olympus, New York, NY, USA). A Texas Red filter cube (562/40 ex., 593, 624/40 em.) was employed to excite dU-BZ and collect the fluorescence in the optimized wavelength range.

## 5. Conclusions

A new deoxyribonucleoside-modified cyanine dye was prepared and characterized. Far-red absorption and emission of this new dye are potentially favorable for *in vitro* and *in* *vivo* imaging to better discriminate the fluorescence signal from autofluorescence and facilitate deep tissue imaging. Viscosity-dependent studies, including fluorescence quantum yields, fluorescence lifetimes, and non-radiative rate constants were determined, and results were in accordance with that predicted by theory for molecular rotors. An impressive 30-fold enhancement in fluorescence intensity in homogenous glycerol/methanol solutions was obtained in a viscosity-dependent manner. Correspondingly, fluorescence lifetimes increased from 0.19 to 1.07 ns with increasing viscosity from 58 to 950 cP. Subsequent *in vitro* investigation suggested that dU-BZ may be capable of functioning as a microviscosity sensor at cellular and subcellular levels. Our results qualitatively support that the dye can readily enter cells and exhibit a modulated fluorescence response in an *in vitro* environment as a function of viscosity, which demonstrates the potential of this new compound as a microviscosity probe at the cellular level. Other factors and quantitative analysis may be considered in future studies.

The new probe, with absorption in the red and emission in the far-red, and viscosity-dependent fluorescence without confounding aggregation or polarity effects, as reported for previous probes [25], is promising for cell culture studies to study the dynamics of cell mitosis where various stages of mitosis are characterized by accompanying changes in viscosity. Red excitation is much less phototoxic than short wavelength visible or UV, thus, should provide valuable information. Additionally, this probe may be useful in the study of mucociliary transport and the dynamics of mucus formation important in various respiratory diseases, such as asthma and cystic fibrosis, in which alterations of the viscoelastic properties of mucus exerts a significant influence on organ function and disease development [29]. Probing the viscosity of mucus not only in cell culture, but also *in vivo*, via bronchoscopy, is particularly intriguing due to the ready accessibility of the lung surface with a red-absorbing and far-red emitting probe. To translate the use of this probe *in vivo*, two-photon excitation may be possible with excitation at *ca*. 1000 nm, an aspect that may be the subject of future studies as deep tissue imaging of up to 1.6 mm has been reported with two-photon fluorescent probes in muscle tissue vasculature [32]. Thus, the newly designed deoxyribonucleoside-modified cyanine dye is a promising candidate as a far-red viscosity sensor for bioimaging.

## Figures and Tables

**Table 1:** Fluorescence quantum yield (Φ_f_) of dU-BZ and viscosity (η) as a function of temperature (T) in 99% glycerol/1% methanol (*v*/*v*) solution.

Temperature (T)/K	Viscosity (η)/cP	Fluorescence Quantum Yield (Φ_f_)
343	50.6	0.04 ± 4 × 10^−3^
333	81.3	0.07 ± 7 × 10^−3^
323	142	0.10 ± 1 × 10^−2^
313	284	0.17 ± 2 × 10^−2^
303	612	0.24 ± 2 × 10^−2^
293	1412	0.34 ± 3 × 10^−2^

**Table 2:** Fluorescence quantum yield (*φ*_f_), fluorescence lifetime (*τ*_f_) *, radiative (*k*_r_) and non-radiative (*k*_nr_) rate constants of dU-BZ as a function of viscosities (*η*) in glycerol/methanol solutions.

% Glycerol	Viscosity (*η*)/cP	Fluorescence Quantum Yield (*Φ*_f_)	Fluorescence Lifetime (*τ*_f_)/ns *	*k*_r_/ns^−1^	*k*_nr_/ns^−1^
95	950	0.26 ± 2.5 × 10^−2^	1.07	0.243	0.692
90	630	0.19 ± 2 × 10^−2^	0.87	0.218	0.931
85	350	0.13 ± 1.5 × 10^−2^	0.59	0.220	1.475
80	250	0.11 ± 1 × 10^−2^	0.57	0.193	1.561
75	170	0.07 ± 7 × 10^−3^	0.40	0.175	2.325
70	130	0.06 ± 6 × 10^−3^	0.30	0.200	3.133
65	70	0.06 ± 6 × 10^−3^	0.28	0.179	3.393
60	58	0.03 ± 3 × 10^−3^	0.19	0.158	5.105
50	28	0.02 ± 2 × 10^−3^	0.10	0.200	9.800
40	13	0.015 ± 1.5 × 10^−3^	0.06	0.250	16.417
30	7.7	0.010 ± 1 × 10^−3^	0.05	0.200	19.800
20	4.8	0.008 ± 8 × 10^−4^	0.04	0.200	24.800
10	1.8	0.006 ± 6 × 10^−4^	0.05	0.120	19.880

* The lifetime resolution of the experimental system is *ca.* 80 ps, with measured lifetimes <0.10 ns estimates only. Lifetimes are ± 0.10 ns.
